# Statistics of Medical Education in the United States

**Published:** 1881-03

**Authors:** 


STATISTICS OF MEDICAL EDUCATION IN THE UNITED STATES.

The Report of the U. S. Commissioner of Education, for the year 1878, contains some interesting statistics regarding the medical, dental, and pharmaceutical schools of the country.

The number of these schools reported to the Bureau during the year was 106. These had 1,337 instructors and 11,830 students. The regular school of medicine and surgery reported 64 institutions (the number now is 69); 915 instructors; 8,279 students; 2,506 graduates; 46,065 volumes in libraries; $1,685-250 in grounds, buildings, and apparatus; $214,347 in productive funds, yielding an income of $13,186; and tuition receipts to the amount of $289,398. The eclectics reported 6 institutions; 51 instructors; 448 students; 211 graduates; 3,000 volumes in libraries; $161,000 in grounds, buildings, and apparatus, and $8,960 receipts from tuition. The homœpathists reported 11 schools; 158 instructors; 1,215 students; 363 graduates; 39,800 volumes in libraries; $349,000 in grounds, buildings, and apparatus, and $95,471 receipts from tuition fees.

The dental schools report as follows: number, 12; instructors, 161; students, 701; graduates, 218; volumes in libraries, 505; value of grounds, buildings, and apparatus, $68,000; receipts from tuition fees, $60,734.

The pharmaceutical schools number 13; instructors, 52; students, 1,187; graduates, 380; volumes in libraries, 5,175; value of grounds, buildings, and apparatus, $155,000; receipts from tuition fees, $25,487.

The medical degrees conferred in course were 3,814; honorary degrees, 4. There were, during the same year, conferred in course 222 degrees in theology; 1,000 in law; 6,367 in arts.

The total amount of educational benefactions during the year is $3,103,289, of which schools of medicine received $8,762. Of this sum, however, regular medical schools received only $4,662, one of these schools being the New York Medical College for Women.

The increase in medical students over the previous year (1877) was 615, the total number being 11,830. The increase of the previous year was 1,082.—*N. Y. Medical Record.*

